# Journal of Medical Internet Research is now indexed in Medline

**DOI:** 10.2196/jmir.3.3.e25

**Published:** 2001-08-28

**Authors:** G Eysenbach

We are very excited and proud to announce that in July 2001 the Journal of Medical Internet Research (JMIR) has been selected by the National Library of Medicine (NLM) to be indexed in Medline/Index Medicus, the world's most important biomedical bibliographical database. NLM uses an advisory committee, the Literature Selection Technical Review Committee, composed of authorities knowledgeable in the field of biomedicine, such as physicians, researchers, educators, editors, health science librarians, and historians, to review and recommend the journal titles NLM should index. Only 15-20% of medical journals are selected at each session for inclusion in Medline, and only very few pure electronic journals have been selected for indexing so far. The Committee recently completed a review of journals for possible inclusion in the National Library of Medicine's database systems and decided to select the "Journal of Medical Internet Research" for indexing and inclusion in Index Medicus and Medline. This will include not only articles which will be published in the future, but also retrospectively all articles which have been published in the Journal in the past 3 years, back to the first issue.

An electronic journal with no print equivalent must meet certain additional criteria to be selected for indexing. First, no restrictions on viewing the journal may exist (other than it might be fee-based). Secondly, NLM should be allowed to make copies of the content to preserve articles for posterity. Thirdly, publishers must provide XML/SGML-tagged data.

The Journal of Medical Internet Research is currently working on the required XML files to make these citations available to NLM. As soon as this process is finished, all articles published in the Journal of Medical Internet Research will be searchable via PubMed and other MEDLINE systems. We will then join the ranks of more than 150 publishers of nearly 1,000 MEDLINE journals (40% of MEDLINE's current citations) who are sending electronic XML data for citations and abstracts for quick inclusion in PREMEDLINE. PubMed will also offer links back to the respective article on the JMIR Web site. In addition, the Journal is working towards inclusion of its full-text articles into PubMed Central.

To date, only 20 pure electronic journals have been chosen for indexing by the National Library of Medicine, plus the BiomedCentral (BMC) specialty journals and the Cochrane database of systematic reviews. Table 1 lists all electronic journals chosen by NLM as of August 2001. Of the 20 electronic journals (excluding BMC and the Cochrane database), only 10, including JMIR, appear to be active. At least 3 electronic journals have been discontinued after a few years, and the remaining 7 journals have not provided any XML-tagged data or have for some other reasons no articles indexed to date.

The editorial board of the Journal of Medical Internet research is proud to be among the pioneers of successful and sustainable online journals and thanks everybody who has contributed to this success, most of all our authors, our peer-reviewers and our readers, who continue to give us useful feedback.

*Gunther Eysenbach*

*Editor,*

*Journal of Medical Internet Research*

**Table 1:** Electronic health journals selected for indexing in MEDLINE by the National Library of Medicine to date (as of Aug 28, 2001), in alphabetical order. Electronic journals were searched using Pubmed's journal browser, by entering the keyword *computer file*. Active journal titles (having published articles in 2001) are printed in bold

Title	No of articles indexed	Active	Issues indexed	**MEDLINE Abbr.** (and link to Pubmed)
AAPS pharmSci [computer file	---			AAPS PharmSci
**BMC journals** (consists of currently 58 specialty journals)	222	**yes**	2000 - today	http://www.ncbi.nlm.nih.gov/entrez/query.fcgi?db=PubMed&cmd=Search&term=%22BMC+Anesthesiol%22[Jour]**BMC Anesthesiol etc.**
CDR weekly [computer file] : communicable disease report	---			CDR Wkly (Online)
**Cochrane database of systematic reviews** (Online : Update Software)	1253	**yes**	2000 - today	**Cochrane Database Syst Rev**
**Dermatology online journal** [computer file].	86	**yes**	1997 - today	**Dermatol Online J**
EJIFCC [computer file] / IFFC	---			EJIFCC
Eurosurveillance weekly [computer file				--
**Frontiers in bioscience** [computer file] : a journal and virtual library.	565	**yes**	1996 - today	**Front Biosci**
Health information systems and telemedicine [computer file	5	no	1995-1996	Health Inf Syst Telemed
Journal of applied clinical medical physics [computer file] / American College of Medical Physics	---			J Appl Clin Med Phys
**Journal of medical Internet research** [computer file].	58	**yes**	1999 - today	**J Med Internet Res**
**Journal of pharmacy & pharmaceutical sciences** [computer file] : a publication of the Canadian Society for Pharmaceutical Sciences, Societe canadienne des sciences pharmaceutiques.	61	**yes**	1998 - today	**J Pharm Pharm Sci**
**MedGenMed** [computer file] : medscape general medicine.	123	**yes**	1999 - today	**MedGenMed**
**Medscape women's health** [computer file].	151	**yes**	1996 - today	**Medscape Womens Health**
**Molecular vision** [computer file].	172	**yes**	1995 - today	**Mol Vis**
Neurology & clinical neurophysiology [computer file] : NCN	---			Neurol Clin Neurophysiol
Neurosurgical focus [computer file	---			Neurosurg Focus
**Online journal of issues in nursing** [computer file].	26	**yes**	2001 - today	**Online J Issues Nurs**
transduction knowledge environment	---			Sci STKE
**Sleep research online** [computer file] : SRO.	67	**yes**	1998 - today	**Sleep Res Online**
The Online journal of current clinical trials [computer file	52	no	1992 - 1996	Online J Curr Clin Trials
The hospitalist [computer file] : the newsletter of the National Association of Inpatient Physicians	18	no	1997 - 1999	Hospitalist

